# Novel Diagnostic Tools for Identifying Cognitive Impairment in Dogs: Behavior, Biomarkers, and Pathology

**DOI:** 10.3389/fvets.2020.551895

**Published:** 2021-01-15

**Authors:** Zuzana Vikartovska, Jana Farbakova, Tomas Smolek, Jozef Hanes, Norbert Zilka, Lubica Hornakova, Filip Humenik, Marcela Maloveska, Nikola Hudakova, Dasa Cizkova

**Affiliations:** ^1^Center for Experimental and Clinical Regenerative Medicine, University of Veterinary Medicine and Pharmacy in Košice, Košice, Slovakia; ^2^Institute of Neuroimmunology, Slovak Academy of Sciences, Bratislava, Slovakia; ^3^Neuroimunology Institute, n.p.o., Bratislava, Slovakia

**Keywords:** canine cognitive dysfunction, neurodegeneration, CADES, questionnaire biomarkers, TAU, Aβ42, NFL

## Abstract

Canine cognitive dysfunction syndrome (CCDS) is a progressive neurodegenerative disorder in senior dogs that is mainly associated with decreased ability to learn and respond to stimuli. It is commonly under-diagnosed because behavioral changes are often attributed to the natural process of aging. In the present study, we used for the first time a comprehensive approach enabling early diagnosis of canine patients with mild cognitive disorders (MiCI). We included **CA**nine **DE**mentia **S**cale (CADES) questionnaires, biochemical parameters, and biomarkers in blood serum, and correlated them with post-mortem histopathological changes. The CADES questionnaires enabled us to identify MiCI dogs developing changes mainly in domains corresponding to social interaction and spatial orientation, which seems to be crucial for delineating early cognitive disorders. Biochemical analyses in these dogs showed slightly elevated liver enzyme parameters (AST and ALT) and significantly decreased sodium and chloride levels in blood serum. Furthermore, we describe for the first time a significant increase of neurofilament light chain (NFL) in blood serum of MiCI dogs, compared to normal aging seniors and young controls, but no changes in TAU protein and amyloid-β (Aβ42) peptide levels. In canine brains with cognitive impairment, amyloid plaques of mainly diffuse and dense types were detected. Furthermore, activated microglia with amoeboid body and dystrophic processes occurred, in some cases with spheroidal and bulbous swellings. On the other hand, no TAU pathology or neurofibrillary tangles were detected. These results suggest that a combination of CADES questionnaire mainly with CNS injury biomarker (NFL) and with biochemical parameters (ALT, AST, Na, and Cl) in blood serum may predict CCDS in senior dogs.

## Introduction

The population of aging dogs is growing significantly, similar to the increase in senior numbers in the human population. Currently dogs live much longer due to the excellent care of their owners, who provide them with a balanced diet, regular exercise and training, dental care, and social interactions, but also regular preventive examinations by veterinarians (1). There is no doubt that prolonged life expectancy is associated with different age-related systemic diseases, which are treated at veterinary clinics as per standard treatment procedure (symptomatology-based and anti-inflammatory treatment) (2). However, senior dogs are also exposed to a higher risk of prevalence of neurodegenerative diseases, for which there is no effective therapy available. Neurodegenerative features in elderly dogs are manifested by cognitive deficits that could be paralleled with human neurodegenerative symptoms, particularly Alzheimer's disease (AD) (3). However, to understand the similarities and differences between canine and human cognitive impairment, there is a need to include approaches supported by reliable cognitive tests, biomarkers, and pathological changes associated with neurodegeneration. We also need to consider the variability in aging between different breeds. In general, large-breed dogs are considered to be seniors at 6–7 years of age, whereas in small breeds, it is not until they reach 10 years (1).

Many studies have documented that senior dogs may experience canine cognitive dysfunction syndrome (CCDS), also known as “canine dementia.” In these dogs, we usually notice behavioral changes that are described in short by the acronym DISHA [**D**isorientation, altered social **I**nteractions, altered **S**leep–wake cycles, **H**ouse soiling and loss of other learned behaviors, altered **A**ctivity levels and increasing Anxiety; (4–8)], as well as neuropathological changes in the brain. CCDS is observed mainly in large-breed dogs over 8 years of age showing slow onset of behavioral and cognitive changes. Several questionnaires are available, which can discriminate between cognitive decline at different stages in senior dogs (7, 9–11). Our group has developed a **CA**nine **DE**mentia **S**cale (CADES) questionnaire, which can distinguish the onset of behavioral and cognitive changes, as well as the severity and progression of cognitive impairment in senior dogs (8). This questionnaire contains 17 items separated into four domains related to changes in behavior, which enable identification of three stages of the disease: mild, moderate, and severe cognitive impairment (8).

However, the key diagnostic tools for neurodegenerative disease in dogs rely on biomarker screening in both blood and cerebrospinal fluid (CSF) (12, 13). Due to the fact that most owners do not agree with CSF collection, blood samples remain as a suitable surrogate for biomarker candidates. So far, there are few studies showing alterations in Aβ_42_ plasma levels between younger healthy, mildly, and severely cognitively impaired dogs (13, 14). Another study showed high CSF amyloid-β (Aβ) levels in younger dogs, which are not likely to harbor deposits of amyloid in their brains yet (15).

Thus, due to some contradictions in Aβ levels, there is a need to include other biomarker candidates such as TAU protein and neurofilament light protein (NFL), firstly studied as blood-based biomarkers in AD (16). In this context, human clinical trials have played an important role in the search for new biomarkers. Both proteins can be analyzed by means of highly sensitive a single-molecule array method (Simoa, Quanterix), which enables detection and monitoring of very low concentrations of biomarkers in blood samples (16). Recently, NFL which is released into the bodily fluids when axons are damaged, has been considered as a potential biomarker for various cerebral proteopathies, including human AD (16, 17). Furthermore, data from clinical study of familiar AD suggest that NFL levels in the blood may serve as a biomarker to monitor neurodegeneration and disease progression already in presymptomatic stage of AD (17).

Despite available behavioral tools at veterinary clinics, owners are not well aware of behavioral changes in their dogs and they often overlook the early onset of CCDS, leading to late diagnosis of CCDS when the brain tissue is conclusively damaged. Particularly in cognitively impaired dogs, reduction in neuron numbers has been described in the cortex, hippocampus, and the limbic system (18, 19). Brain atrophy is often linked to multifaceted pathologies such as enlargement of lateral ventricles, dysfunction of the glymphatic and meningeal lymphatic systems, changes in the choroid plexus, vascular changes, significant increase in protein accumulation such as TAU protein, α2-macroglobin, amyloid peptides, albumin or transthyretin in CSF (20), damage to DNA, and inflammation mainly in prefrontal cortex (14, 21). However, the main concern is associated with formation of large aggregates of Aβ deposits leading to structural damage in brain tissue (22). These aggregates spread in a prion-like manner, forming intracellular and extracellular deposits that impair the proper functioning of neurons. There is evidence of Aβ deposits and amyloid angiopathy (microangiopathy and macroangiopathy) in the brain of aged dogs (22, 23). Besides Aβ plaques, inflammatory response with activation of microglial cells and astrocytes has also been noticed in dogs with CCDS (microgliosis and astrocytosis) (23, 24). Unlike Aβ deposits, TAU neurofibrillary inclusions have only rarely been identified in affected canine brains (24). This seems to be so far an important pathological difference of neurodegeneration between human (severe TAU pathology) and canine tissue (rare TAU pathology). Furthermore, neural death leads to decrease in excitatory neurotransmitters such as acetylcholine with its neuronal projections from basal forebrain to cerebral cortex and hippocampus, a component of cognitive function, especially memory; dopamine in the substantia nigra in the midbrain, ventral tegmental area, and hypothalamus, involved in motoric function; norepinephrine in the pons, related to waking and attention; and serotonin in the brainstem, controlling mood, and sleep cycle. These neurochemical changes lead to neurotransmitter system dysfunction, together with reduced neuronal and synaptic function, which is seen in aging dog brains and may be responsible for the clinical expression of CCDS (10).

The aim of our study was to stratify canine patients with mild CCDS (MiCI), characterized by slow onset of clinical symptoms. In this case, we used for the first time a comprehensive approach involving four components: (i) clinical and neurological observation in order to exclude other concurrent diseases with similar symptomatology as CCDS, (ii) behavioral scoring involving CADES for monitoring early behavioral changes, (iii) monitoring of neurodegenerative biomarkers (NFL, TAU protein, and Aβ42 peptide) in blood samples, and (iv) analyzing pathological changes in post-mortem dog tissues. Our data confirmed that these tools are reliable in capturing early cognitive changes and can be used for early diagnosis of CCDS in elderly dogs. Early diagnosis can differentiate pathological aging from the normal aging process in order to begin early treatment. This will increase the chances to extend dogs' life in good cognitive and physical condition and to prolong a healthy human–dog relationship. So far, the recommended treatment lies only in antioxidant nutritional supplements such as denamarin, silybin, vitamin E, cholodin, and omega-3 fatty acids that can be added to any diet.

## Materials and Methods

### Dogs

We have selected suitable dogs for our study, from patients who came for regular preventive examinations to our University Hospital of UVMP. From the wider group of 40 dogs over 8 years of age, 30 owners agreed with participation on the study, and from them, we selected only 24 dogs that did not suffer from any secondary diseases. As for young dogs, we obtained approval from 15 dog owners. Due to the owner's checkout, one dog was excluded from this group.

We examined 30 senior dogs of 14 different medium breeds, over 8 years old, and 15 young dogs of 7 different medium breeds, <3 years old, both sexes with body weight ranging between 10 and 20 kg. These dogs were selected from regular patients visiting the University Veterinary Hospital at the University of Veterinary Medicine and Pharmacy in Kosice, Slovakia during 1 year. All animals were treated according to European legislation on animal handling and experiments (86/609/EU). We adhered to a high standard of veterinary care to ensure that all studied dogs received the best care available. A separate group of senior dogs (*n* = 5) aged 13–14 years old, of various breeds and weighing 10–15 kg, was analyzed with CADES scores and afterwards euthanized due to various fatal diseases and used for pathological analyses. Two additional dogs were used for positive control (13-year-old Pekingese, 8 kg, CADES 27) and negative control (9-year-old American Cocker Spaniel, 11 kg, CADES 0). Dogs were on no medication, the body weight (10–20 kg) correlated with the standards for corresponding breed, and body condition score was 4–5, according to World Small Animal Veterinary Association (WSAVA) standards. For all dogs included in our study, we obtained fully informed consent from the dog owners.

### Clinical and Neurological Observation

All dogs were processed for clinical and neurological examination (including evaluation of mentation, posture and gait, cranial nerves, postural reaction, spinal reflexes, pain, and spinal palpation), urine examination [pH, protein (Dipstick), protein-to-creatinine ratio, glucose, ketones, and bilirubin], complete blood count (CBC), and serum chemistry screening [selected biochemistry parameters—aspartate aminotransferase (AST), alanine transaminase (ALT), alkaline phosphatase (ALP), gamma glutamyl transferase (GMT), pancreatic amylase (pAMS), lipase (LPS), creatinine (CREA), UREA, total protein (TP), albumin (ALB), ammonium (NH3), glucose (GLU), cholesterol (CHOL), triglyceride (TG), total lipids (TL), phosphor (P), calcium (Ca), chloride (Cl), natrium (Na), and kalium (K)], and thoracic and abdominal ultrasound examination was performed to exclude tumors. All blood serum samples immediately underwent blood chemistry examination, and the rest of serum samples were frozen at −80°C until use. Furthermore, we performed other necessary examinations (ophthalmology, orthopedic, and x-rays) in order to exclude other concurrent diseases with similar symptomatology to CCDS. Finally, after excluding patients with systematic diseases, we selected 24 senior dogs (S) (>8 years) and 14 young control dogs (YC) (<3 years), which were assigned to our study with the owners' approval.

### Behavioral Scoring for Cognitive Impairment

For clear distinction between physiological and pathological aging, we used the canine dementia scale (CADES) questionnaire developed by our group (8). Experienced veterinarians filled in this questionnaire together with the owners, while observing the dogs to see if any abnormal behavior appeared. The CADES questionnaire is divided into four domains reflecting clinical signs of CCDS. These domains are A: spatial orientation, B: social interaction C: sleep–waking cycle, and D: house soiling. Every domain consists of a couple of items. In general, CADES comprises 17 items all scored from 0 to 5 points; 0 points means no abnormal behavior was observed; 2, abnormal behavior was detected at least once in 6 months; 3, abnormal behavior was detected at least once in a month; 4, abnormal behavior appeared two to four times in 1 month; 5, abnormal behavior was seen several times a week. Number 1 is excluded from the scale as it brings easier scoring from 0 to 95 as a total score from 17 items in four domains (A+B+C+D). The total score (0–95) from the questionnaire can divide patients into four stages according to clinical manifestation of behavioral changes. For normal aging, the score is 0–7 points; mild cognitive impairment, 8–23 points; moderate cognitive impairment, 24–44 points; and severe cognitive impairment, 45–95 points (Supplementary Table 1, CADES).

### Collection of Blood Samples

Fresh blood samples (5–6 ml/dog) were collected by experienced veterinarians from senior dogs (*n* = 24, normal aging dogs NA: *n* = 11; mild cognitive impairment dogs MiCI: *n* = 13), and young control dogs (YC: *n* = 14) from the cephalic vein, and were collected in serum-separator tubes (4.4 ml, 75 × 13 mm, Z-Gel, Sarsted AG & Co KG, Nümbrecht, Germany) and in tubes containing anticoagulant (K3E 1.3 Micro tube, 1.6 ml EDTA/ml, Sarsted AG & Co KG, Nümbrecht, Germany). The tubes were allowed to stand for 15–30 min at room temperature and afterwards were centrifuged for 10 min at 3,500 × g. Separated serum was collected, apportioned into 1.0-ml aliquots, and stored at −80°C until use for additional analyses.

### Screening Biomarkers Related to Neurodegeneration

Concentrations of neurofilament light chain (NFL), TAU proteins, and Aβ42 peptide in blinded dog serum samples were measured using Simoa digital ELISA, using an HD-1 Analyzer (Quanterix, Boston, USA). For the analysis, Simoa™ NF-Light Advantage Kit, Simoa™ Mouse TAU Discovery Kit, and Simoa™ Aβ-42 Advantage Kit were used (Quanterix, Boston, USA). Briefly, frozen dog serum samples were melted, centrifuged for 10 min at 25°C, and 20,000 × g, and supernatants were transferred onto Simoa sample plates together with calibrators for NFL, mouse TAU, and Aβ42 kits (parts of kits). Each measurement was done in duplicate according to the manufacturer's recommendations. Concentrations were calculated using Simoa™ HD-1 instrument software.

### Post-mortem Neurodegenerative Changes in Brain Samples

#### Brain Samples

Senior dogs (*n* = 5) aged 13–14 years old, of various breeds and weighing 10–15 kg, were separated into two groups, one with CADES scores 10, 17, and 23 (*n* = 3) and the other with CADES scores 39 and 63 (*n* = 2), and were then euthanized due to various fatal diseases. Two additional dogs were used for positive control (13-year-old Pekingese, 8 kg, CADES 27) and negative control (9-year-old American Cocker Spaniel, 11 kg, CADES 0). A set of archived brain samples from aged dogs diagnosed antemortem with CADES without matched serum samples were used for additional immunohistochemical analyses. Dogs with brain lesions such as neoplasms and vascular lesions, or with inflammatory diseases confirmed either physiologically or histopathologically, were excluded from the study. No other organs except brain were examined histologically to rule out other causes of neurological signs. Routine necropsies were performed at the Department of Anatomy of the University of Veterinary Medicine and Pharmacy in Kosice, after obtaining informed consent from the dog owners. The canine brains were removed from the skull and postfixed in 4% PFA for several weeks.

#### Immunohistochemistry (IHC) on Paraffin Sections

Canine brains were postfixed in 4% paraformaldehyde (PFA), without sucrose gradients. After fixation, brains were dissected and the frontal cortex and hippocampus were straight processed in automated vacuum tissue processor (Leica, ASP 300), embedded in paraffin tissue blocks, and later cut on a microtome (Leica RM2255) into 8-μm paraffin-embedded sections. IHC staining was preformed manually by experienced technicians according to standardized protocol. These sections were treated with formic acid (98%) and heat pre-treatment, followed by incubation with primary antibodies (Table 1) against tau (1:1,000, AT8, Thermo Scientific, USA), β-amyloid (1:500, 4G8, Covance, USA), and Iba1 (1:500, Wako, USA). All sections were incubated with biotinylated secondary antibody (Vectastain Elite ABC Kit; Vector Laboratories, Burlingame, CA, USA) at room temperature for 1 h and then reacted with avidin-biotin-peroxidase complex for 1 h. The immunoreaction was visualized with VIP (Vectastain Elite ABC Kit; Vector Laboratories, Burlingame, CA, USA) and counterstained with methyl green (Vector Laboratories, CA, USA).

**Table 1:** Antibodies used for IHC.

**Antibody**	**Species raised/clonality**	**Description of immunogen**	**Source**	**Dilution**
Anti-Iba1	Rabbit/polyclonal	Synthetic peptide corresponding to C-terminus of Iba1	Wako Chemicals, USA	1:500
Anti-human PHF-tau (clone AT8)	Mouse/monoclonal	Phospho-PHF-tau pSer2021Thr205	Thermo Scientific, USA	1:1,000
Anti-β-amyloid (clone 4G8)	Mouse/monoclonal	This antibody is reactive to amino acid residues 17–24 of β-amyloid; the epitope lies within amino acids 18–22 of β-amyloid (VFFAE)	Covance, USA	1:500

### Statistical Analysis

The blood serum biochemical parameter data were subjected to analysis of variance (ANOVA) followed by *post-hoc* Tukey analysis to assess differences between the groups (YC, NA, and MiCI). The significance levels were set at ^*^p < 0.05 (significant) and ^**^p < 0.01 (very significant). The Sigma Plot statistical software package (version 13.2; Systat Software Inc., Chicago, USA) was used for data processing. Statistical analyses of biomarker (NFL, TAU protein, and Aβ42 peptide) concentrations in blood serum were performed by Student *t*-test, and correlation analysis between the CADES score and levels of NFL in serum was assessed using Spearman correlation analysis (GraphPad Prism software, version 8.3).

## Results

### Biochemistry Parameters in Canine Blood

#### Senior Dogs

After blood testing, we found that the patients did not show any remarkable abnormalities in CBC. However, the blood biochemistry results revealed some changed values, and we noticed significant elevation of alanine aminotransferase (ALT), gamma glutamyl transferase (GMT, also known as GGT), and total protein (TP) in MiCI dogs when compared to YC and NA group, although the levels of these parameters were still in the physiological range. Furthermore, we detected significantly decreased values of chloride and sodium in MiCI dogs, below the physiological range, when compared to NA and YC groups. Overall, changes in CBC showed correlation with the cognitive stage according to CADES only in a few parameters (Table 2).

**Table 2:** Blood serum biochemical parameters.

**Blood serum parameter**	**Reference range**	**YC**	**NA**	**MiCI**
AST (μkat/L)	<0.6	0.39 ± 0.11	0.38 ± 0.15^*^	0.54 ± 0.05^*^
ALT (μkat/L)	<0.949	0.52 ± 0.18^**^	0.53 ± 0.19^**^	0.9 ± 0.2^**^^**^
ALP (μkat/L)	<1.24	0.8 ± 0.37	0.61 ± 0.21	0.63 ± 0.22
GMT (μkat/L)	<0.14	0.09 ± 0.05	0.05 ± 0.03^*^	0.13 ± 0.03^*^
Pams (μkat/L)	<7.21	5.95 ± 0.68	5.64 ± 1.2	6.77 ± 1.69
LPS (μkat/L)	<1.66	0.87 ± 0.2	1.15 ± 0.82	0.68 ± 0.34
CREA (μmol/L)	46–88	70.24 ± 11.08	65.85 ± 22.18	73.05 ± 24.86
UREA (mmol/L)	3.97–8	5.23 ± 1.62	4.08 ± 1.02	4.24 ± 2.23
TP (g/L)	47–74	52.37 ± 5.31^*^	61.55 ± 2.39^*^^*^	63.4 ± 9.13^*^
ALB (g/L)	26–41	33.64 ± 2.69	33.9 ± 4.19	36.02 ± 2.2
NH3 (μmol/L)	<47	13.87 ± 2.93	19.4 ± 14.42	20.11 ± 6.4
GLU (mmol/L)	3.6–5.8	4.44 ± 0.75	4.79 ± 0.46	4.94 ± 0.38
CHOL (mmol/L)	3.3–7.4	5.53 ± 0.73	6.24 ± 2.88	4.6 ± 0.96
TG (mmol/L)	0.3–1.2	0.69 ± 0.3	0.89 ± 0.55	1.03 ± 1.2
TL (g/L)	4.7–7.25	6.14 ± 0.67	6.56 ± 3.4	5.72 ± 1.91
P (mmol/L)	0.9–1.9	1.41 ± 0.3	1.23 ± 0.28	1.06 ± 0.11
Ca (mmol/L)	2.05–2.9	2.5 ± 0.19	2.47 ± 0.13	2.29 ± 0.22
Cl (mmol/L)	110–130	114.57 ± 1.94^**^	112.15 ± 2.27^*^	106.54 ± 6.16^**^^*^
Na (mmol/L)	143–151	145.41 ± 2.21^**^	142.05 ± 2.49^*^	135.48 ± 6.94^*^^*^
K (mmol/L)	3.5–5.1	4.33 ± 0.5	4.35 ± 0.41	4.33 ± 0.4

*Mean ± standard deviations of blood serum parameters evaluated in YC (n = 7), NA (n = 7), and MiCI (n = 7) dogs*.

* *p <0.05 (significant)*,

** *p < 0.01 (very significant)*;

* (YC vs NA),

^*^(YC vs. MiCI),

* (NA vs. MiCI).

#### Young Controls

Blood samples were analyzed for the same biochemistry markers as for senior dogs. In all YC, we observed physiological values of all measured parameters (Table 2).

### Behavioral Analysis

Standardized behavioral testing (CADES questionnaire) was performed on a subset of senior dogs (*n* = 24) in order to distinguish the onset of behavioral and cognitive changes (MiCI) from normal aging (NA). CADES testing helped us to find some behavioral changes by comparing the actual cognitive condition of old dogs to their “top” cognitive condition, which is considered as reached in the age 1–7 years (8). Dogs with more than one owner (shelter dogs adopted in adult age and dogs that had changed owners during their lives) were excluded from the study.

#### MiCI Dogs

**Figure 1:**
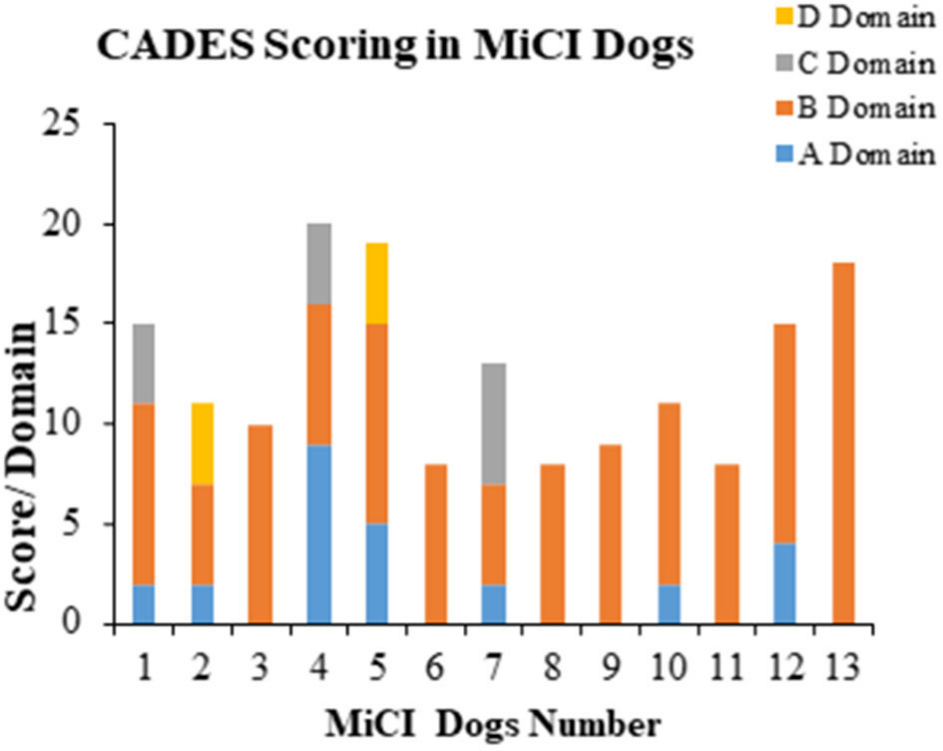
Behavioral analyses of MiCI dogs. Stacked bar graph shows behavioral changes in most affected domains B and A, and occasionally affected domains C and D in 13 dogs with mild cognitive impairment (MiCI) tested with CADES.

We identified 13 senior dogs with mild cognitive impairment according to CADES (score 8–23), showing behavioral changes such as disrupted owner–pet relationship and irritability. The changes in the most affected domain B, which was related to social interaction with owners and other dogs, were identified in all MiCI dogs (Figure 1). The most frequently marked questions were B6 [changes in interactions human–dog and dog–other dog (playing, petting, and welcoming); *n* = 12/13], B7 (changes in individual dog behavior, exploration activity, playing, and performance; *n* = 8/13), and B9 (irritability; *n* = 8/13). Besides B domain, we noticed A domain as the second most affected one, although only seven dogs were identified. Changes in A domain corresponded to spatial orientation, related with items such as abnormal response to familiar objects (A3, *n* = 5/13), aimless wandering (A4, *n* = 2/13), or recognition of familiar people and animals inside or outside (A2, *n* = 1/13). In contrast, much less frequent changes in C (sleep–wake cycle, *n* = 3/13) and D domains (house soiling, *n* = 2/13) were detected. Furthermore, in B domain, we documented the highest scoring (mean 9 ± 3.2) among all domains (A, mean 2.0 ± 2.6; C, mean 1.0 ± 2.1; D, mean 0.6 ± 1.5). Our overview of results observed for MiCI dogs is presented in Figure 1.

#### NA Dogs

In normal aging seniors (*n* = 11), we observed only slight behavioral changes, with low scores affecting domain B–social interaction. Changes were identified in similar items as in MiCI: B6 (*n* = 2/7), B7 (*n* = 2/7), and B9 (irritability; *n* = 1/7), and B8 (response to commands and ability to learn new tasks; *n* = 1/7).

#### YC Dogs

We chose young adult dogs aged <3 years. All young control dogs underwent the whole neurological examination process as the geriatric dogs over 8 years. CADES testing was excluded from examination of YC, as CADES is primarily focused on geriatric dogs. In addition, YC were behaviorally examined to exclude patients with any behavioral problems (dogs aggressive toward people or other dogs, with excessive or compulsive behavior, or with anxiety of any origin).

### Blood Biomarkers for Neurodegeneration

In human blood, levels of NFL and TAU protein can distinguish patients with several different neurodegenerative conditions from healthy ones, so we also used these two biomarkers for screening of blood serum from dogs enrolled in our study (MiCI, NA, and YC). In addition, we also analyzed Aβ-42 peptide in blood serum, because Aβ aggregates were detected in dogs with CCDS (24). We measured levels of NFL, TAU protein, and Aβ-42 peptide in canine serum using Simoa technology (Quanterix).

**Figure 2:**
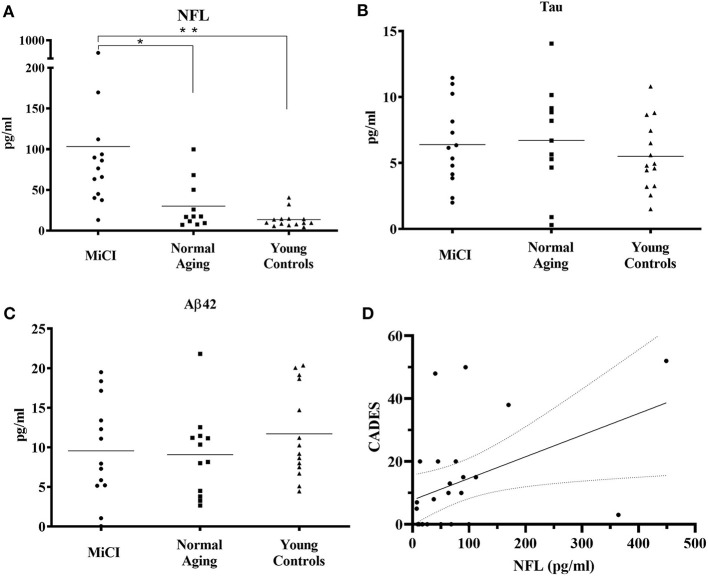
Analysis of NFL, TAU protein, and Aβ-42 peptide levels in canine serum. Analyses were performed using single molecule array (Simoa) digital ELISA assay. Concentrations of NFL **(A)** in canine serum were 103.3 ± 111.1 pg/ml for MiCI dogs, 30.2 ± 30.0 pg/ml for NA dogs, and 13.7 ± 10.4 pg/ml for YC dogs. NFL levels were 3-fold higher in MiCI dogs (mean 103.3 pg/ml) compared to NA dogs (mean 30.2 pg/ml) with statistical significance (*p = 0.0463) and 7-fold higher compared to YC dogs (**p = 0.0059). TAU protein concentrations **(B)** in canine serum were 6.4 ± 3.1 pg/ml for MiCI dogs, 6.7 ± 4.0 pg/ml for NA dogs, and 5.5 ± 2.7 pg/ml for YC dogs. Concentrations of Aβ-42 peptide in canine serum **(C)** were 9.6 ± 6.3 pg/ml for MiCI dogs, 9.1 ± 5.4 pg/ml for NA dogs, and 11.7 ± 5.7 pg/ml for young control dogs. Note a significant correlation between CADES score and NFL levels in blood serum of analyzed MiCI and normal aging dogs (*p* < 0.0107, Spearman coefficient *r* = 0.4578, 95% confidence interval 0.06418–0.7281) **(D)**.

This analysis showed that NFL levels in the serum of MiCI dogs (mean 103.3 pg/ml) were statistically significantly increased 3-fold (*p* = 0.0463) compared to NA dogs (mean 30.2 pg/ml) and 7-fold increased (*p* = 0.0059) compared to YC dogs (mean 13.7 pg/ml) (Figure 2A). On the other hand, we could not see any differences in serum concentrations of TAU protein between all three tested groups, whereby TAU concentrations in all three tested groups were very similar (MiCI 6.4 pg/ml, NA 6.7 pg/ml, and YC 5.5 pg/ml) (Figure 2B). The same observation was also made for levels of Aβ-42 peptide in canine serum, with measured concentrations at comparable levels (MiCI 9.6 pg/ml, NA 9.1 pg/ml, and YC 11.7 pg/ml) (Figure 2C). Furthermore, we found a significant correlation between CADES score and NFL levels in the blood serum of all analyzed MiCI and normal aging dogs (*p* < 0.0107, Spearman coefficient *r* = 0.4578, 95% confidence interval 0,06418–0.7281) (Figure 2D).

### Immunohistochemical Analysis of Neurodegeneration in Canine Brain

#### Diffuse and Compact Amyloid Distribution

**Figure 3:**
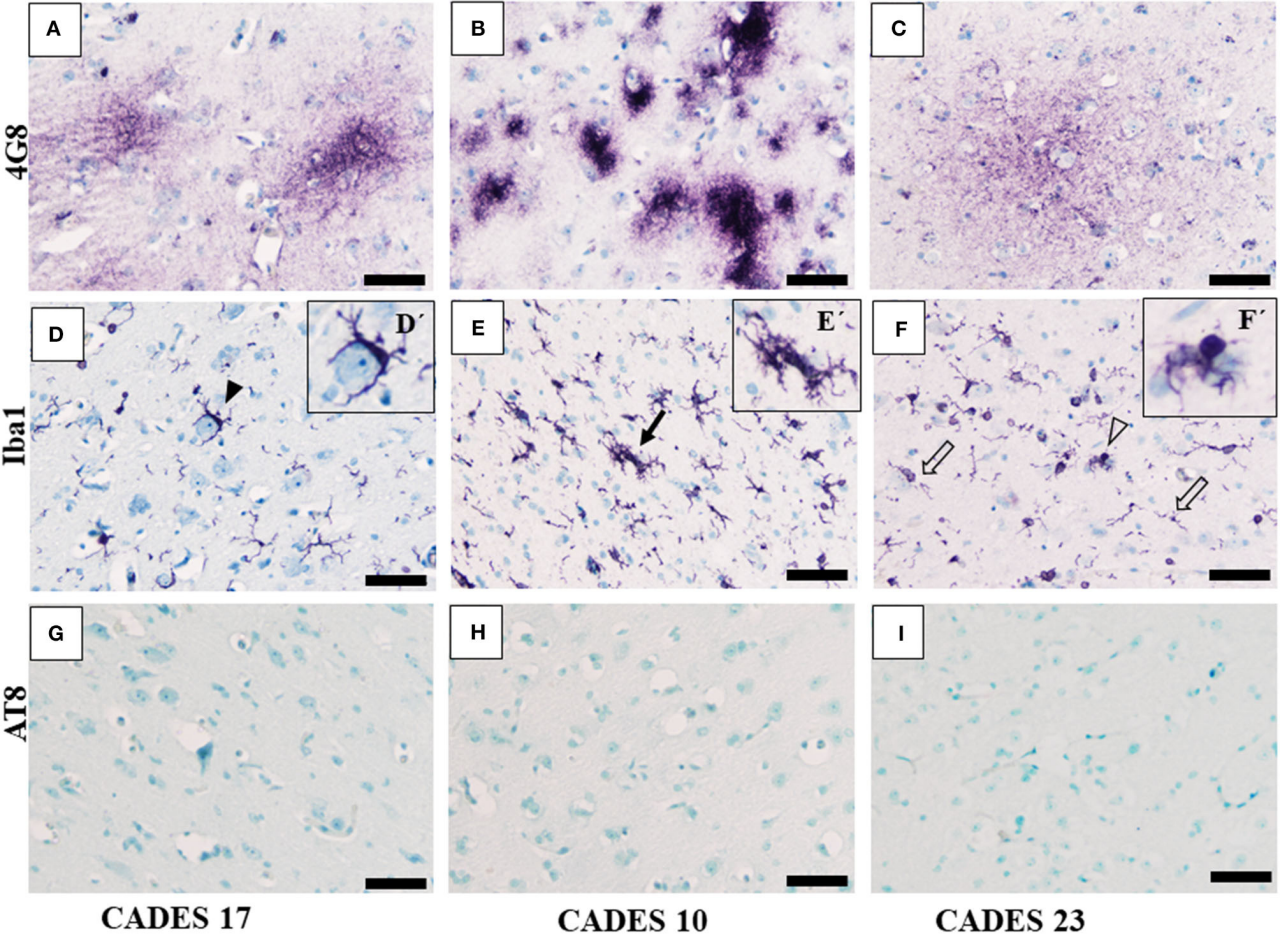
Immunohistochemistry of frontal cortex in MiCI dogs. IHC staining of frontal cortex showed three different types of amyloid deposits/plaques positive for 4G8 antibody **(A–C)** diffuse cloud-like plaques **(A)**, very dense compact plaques **(B)**, and more diffuse plaques **(C)**. Morphology of microglia positive for Iba1 antibody varied among the analyzed brains. Note: the following types of microglia were identified: activated (**D**, full arrowhead), clustered (**E**, full arrow), dystrophic with fragmented processes (**F**, empty arrow), and bulbous swelling (**F**, empty arrowhead). We did not observe any TAU pathology or AT8-positive inclusions in the tested MiCI brains **(G–I)**. Scale bar **(A–F)** 50 μm. Scale bar (D'–F') 20 μm.

**Figure 4:**
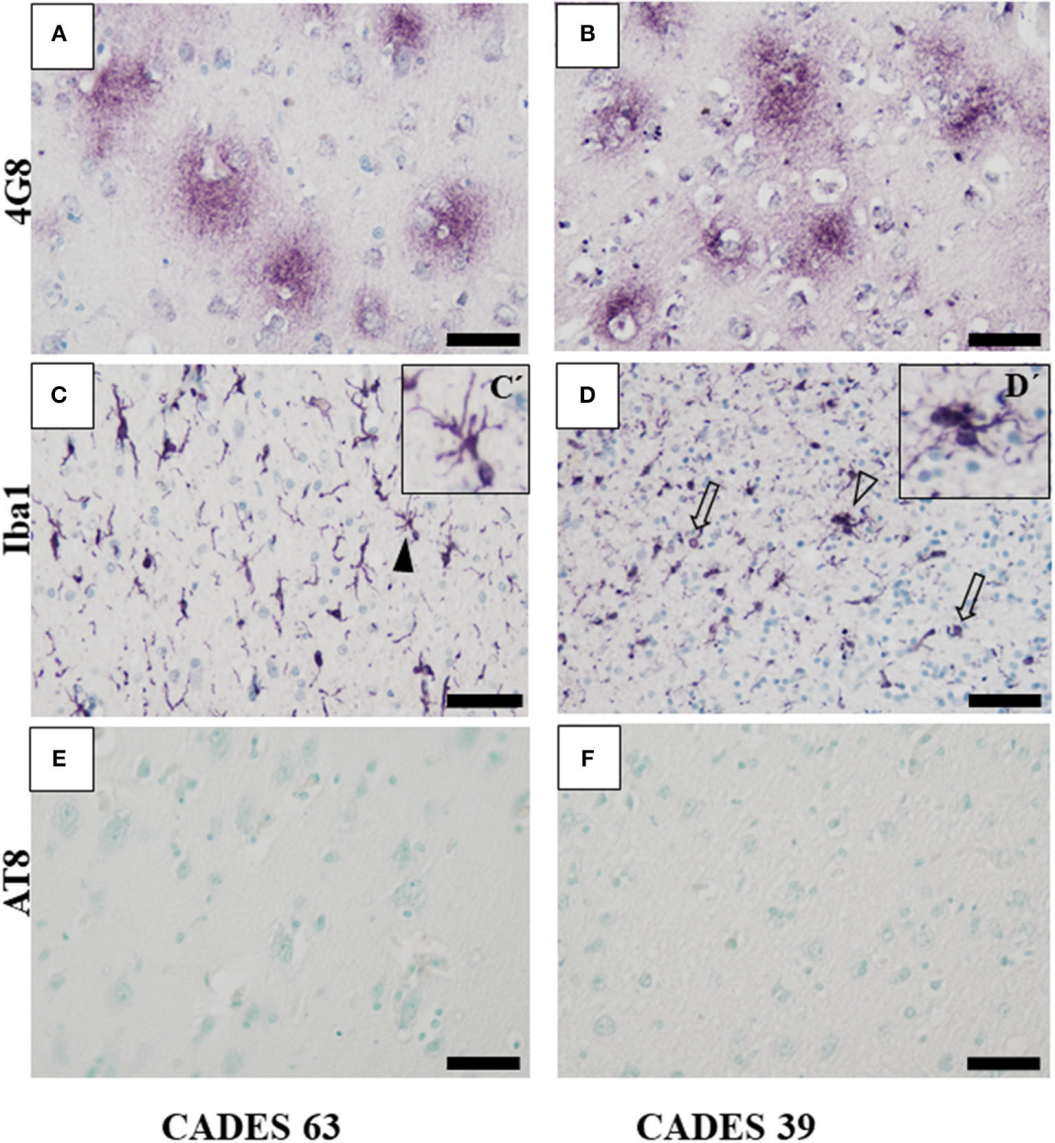
Immunohistochemistry of frontal cortex in CCDS dogs. In both analyzed brains, IHC staining against β-amyloid showed presence of typical diffuse plaques **(A,B)** but no presence of TAU inclusions or AT8 positivity **(E,F)**. Microglia cells positive for Iba1 antibody showed activated state with the following morphology: thicker processes (**C**, full arrowhead, detail in C'), cluster appearance (**D**, empty arrowhead, detail D'), and bulbous structures (**D**, empty arrows). Scale bar **(A–F)**: 50 μm. Scale bar (C', D'): 20 μm.

**Figure 5:**
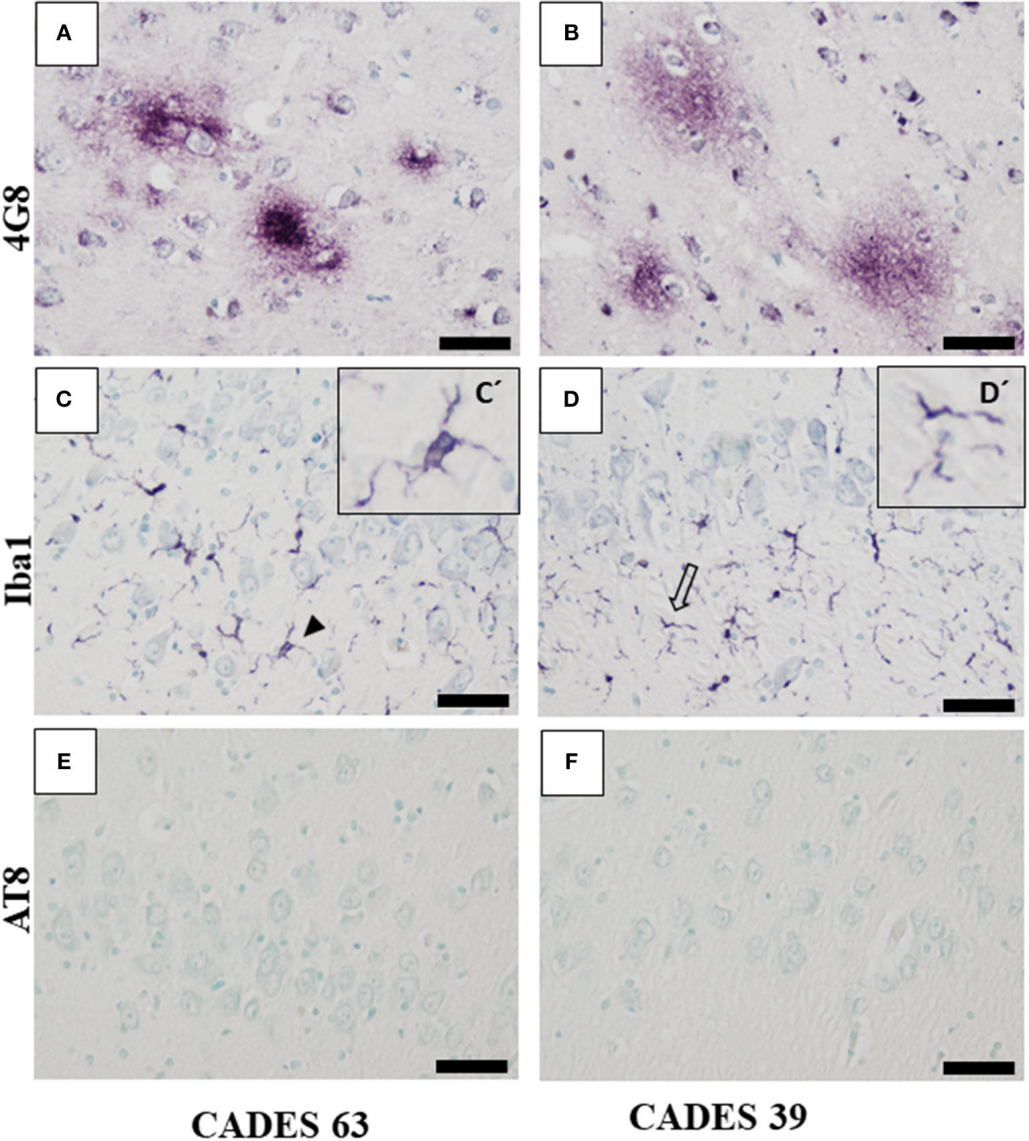
Immunohistochemistry of hippocampus in CCDS dogs. Staining against amyloid beta with 4G8 antibody showed condensed form of amyloid plaques **(A)** in the case with higher CADES score compared to the one with lower CADES score **(B)**, where we observed the typical diffuse form of amyloid plaque. Iba1-positive microglia showed activated (**C**, full arrowhead) and dystrophic morphology (**D**, empty arrow, detail in D') in both cases in CA region **(C,D)**. Note: absence of positive TAU inclusion and AT8 positivity **(E,F)**. Scale bar **(A–F)**: 50 μm. Scale bar (C', D'): 20 μm.

Canine APP (amyloid precursor protein) is 98% homologous with human APP and has the same amino acid sequence (25). In our post-mortem analysis for the presence of amyloid deposits in the dogs' brain tissue, we used 4G8 antibody, which is commonly used for amyloid deposit detection in human post-mortem studies as well (26). Our immunohistochemical study of the frontal cortex (Figures 3, 4) and hippocampus (Figure 5), the brain areas responsible for cognitive behavior, showed the presence of amyloid deposits in typical plaque form. Interestingly, amyloid plaques in brain tissues were also found in three dogs with lower CADES score (<24) in diffuse form (Figures 3A,C), similarly as observed in the normal aging human brain (26). In two individuals with higher CADES scores (>39) and CCDS symptoms, amyloid plaques were found with dense and compact form (Figures 4A,B).

#### Neuroinflammation and TAU Pathology

**Figure 6:**
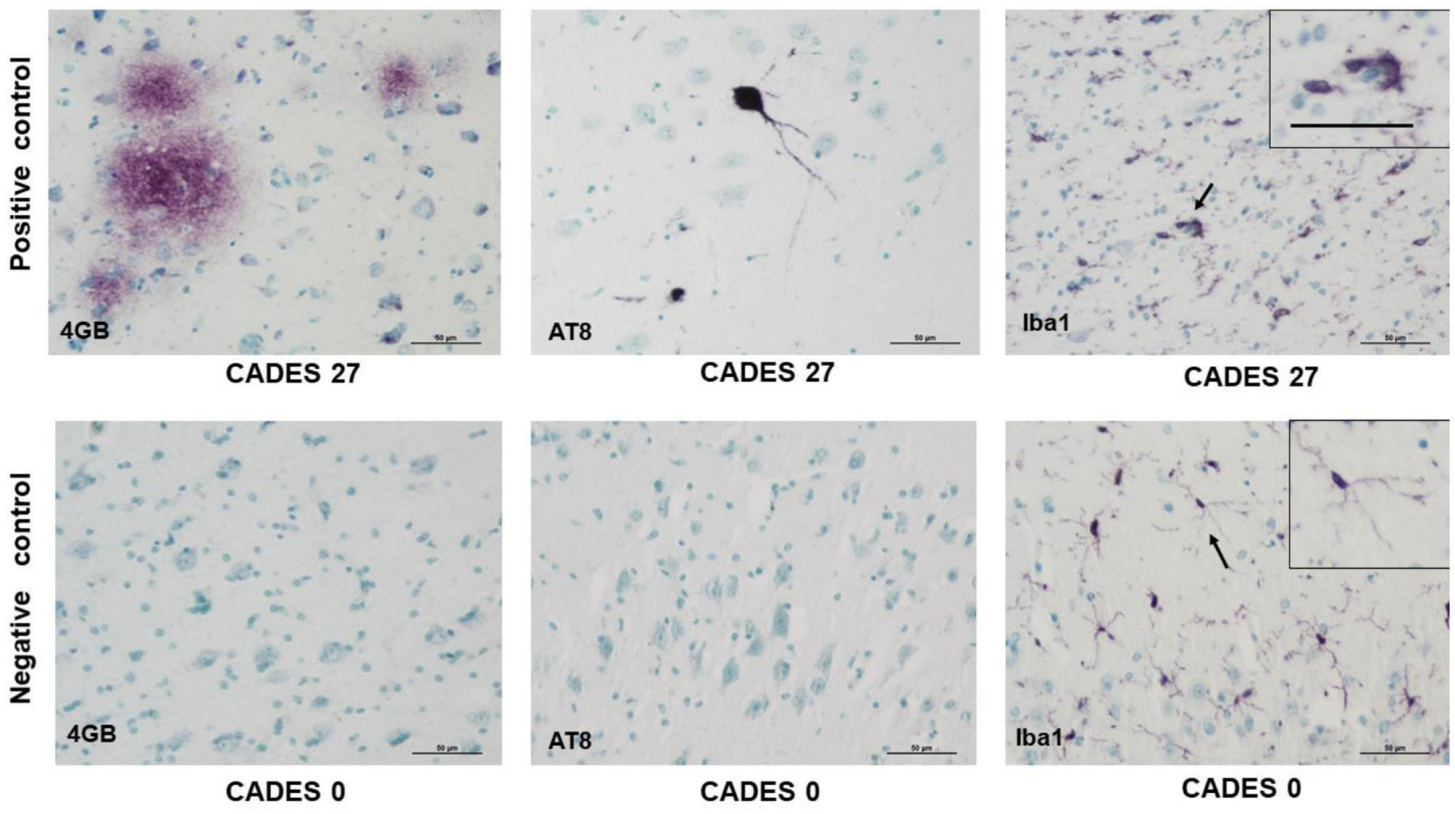
Immunohistochemistry of cortex in positive control/dog with cognitive declaim (CADES 27) and negative control/dog with normal cognition (CADES 0) showed differences in all three antibodies. While in positive control, we could identify amyloid deposits (4GB), occasional TAU (AT8) pathology, and activated microglia (Iba1), with amoeboid morphology (indicated by arrow), cortex of negative control was free of amyloid and TAU. Resting microglia with fine, ramified processes was detected (indicated by arrow). Note, inserts in positive/negative control with higher magnification of microglia morphology. Scale bars: 50 μm.

Neuroinflammation in the examined dogs was represented by reactive and senescent microglia, mainly in the frontal cortex, but also present in the hippocampus. Microglia were presented in various stages and morphological shapes: from phagocytic amoeboid form with characteristic enlarged cell processes (full arrowhead, Figure 3D) to dystrophic with spheroidal or bulbous swellings (empty arrowhead, Figure 3F), sometimes localized in clusters (full arrow, Figure 3E). These findings are similar to those in our previous work (24). In all examined cases, we did not observe any TAU pathology or neurofibrillary tangles positive for AT8 antibody in the frontal cortex or hippocampus. Taken together, we confirmed the presence of β-amyloid plaques and inflammation in three post-mortem brains (Figure 3) with MiCI (mild cognitive impairment) and lower CADES scores, and in two cases with CCDS with higher CADES scores (Figures 4, 5). Positive and negative control showed differences in all three antibodies. While in positive control (CADES 27) we could identify amyloid deposits, occasional TAU pathology, and activated microglia, in negative control (CADES 0), tissue was free of amyloid and TAU, and only resting microglia was detected (Figure 6).

## Discussion

In the present study, for the first time we used a comprehensive approach that could enable early diagnosis of canine patients with mild cognitive disorders (MiCI) involving CADES questionnaires, peripheral biomarkers (AST, ALT, Na, and Cl), and biomarkers for neurodegeneration (NFL, TAU protein, and Aβ42 peptide) in blood serum. In addition, in a separate group of dogs with a CADES score for MiCI, we observed neurodegenerative changes such as amyloid plaque formation and inflammatory changes associated with activated microglia.

A shift toward the diagnosis of early cognitive disorders in humans has emerged due to three main factors: a long pre-dementia stage in neurodegenerative diseases, improvement in early diagnosis methods, and increased emphasis on early intervention to prevent or delay dementia (27). Similarly, these factors play a key role in neurodegenerative diseases in senior dogs (1).

CADES questionnaires, which disclose cognitive decline at different stages in senior dogs, enabled us to identify 13 dogs with mild cognitive impairment (score 8–23). These MiCI dogs revealed changes either only in domain B (social interaction) or in combination with A (spatial orientation), and rarely with C (sleep–wake cycle) and D (house soiling) domains. Thus, changes in social interaction and particularly in spatial orientation seem to be crucial for indicating early cognitive disorders. B domain related to social interactions showed changes in human–dog or dog–other dog interactions. Furthermore, we documented the highest scoring in B domain among all the domains evaluated (9 ± 3.2). However, we also noticed irregularity in the way each domain was scored due to behavioral changes observed in each dog. Occurrence and frequency of specific behavior varied from dog to dog. The high degree of behavioral variety could be explained by suspected differences in neuropathology. The neuropathology of CCDS that fits the clinical picture is well-known, but it varies from case to case, though there are predilection areas in the brain that are responsible for changed behavior. The hippocampus together with the neo-cortex and the amygdala are areas of the brain that play a key role in *memory*, and neuron loss in these areas leads to disorientation, forgetting commands, and house-soiling problems (19). Glial (astrocytic and microglial) alteration may influence the dopaminergic system, which is relevant for animal behavior such as increased aggression, irritability, loss of interest in playing with owners or other dogs, and individual behavior changes (28). Abnormality in the sleep–waking cycle might be reflected in the altered autonomic balance in older dogs (29).

Analysis of MiCI dog blood sera revealed several changed values compared to normally aging dogs, particularly in liver enzymes, which may indicate the beginning of liver dysfunction. We noticed elevation of ALT and AST, liver enzymes essential for amino acid metabolism, and of GMT, an enzyme necessary for the metabolism of drugs and toxic substances. Although all three enzymes were significantly elevated in MiCI dogs compared to the NA group, they reached only the upper limit of reference values, thus still remaining within the physiological range. Blood levels of ALT and AST alike are also used in human clinical practice to measure factors associated with cardiovascular and metabolic diseases, known as risk factors of AD and cognitive decline (30–33). Thus, our data may point to a possible link between peripheral biomarkers of liver functioning (AST and ALT) and the central biomarkers related to AD, including NFL, Aβ42 peptide, and TAU protein and neurodegeneration. However, additional studies are needed to determine whether the involvement of metabolic disturbances in the pathophysiology of cognitive disorders represents a causative or secondary factor, and whether liver enzymes could be enrolled as biomarker candidates for early diagnostics of neurodegeneration (32).

Furthermore, our data clearly show a significant decrease in sodium and chloride levels, below the physiological range in MiCI dogs compared to NA. Hyponatremia can be caused by several factors such as changes in water balance related to age, medications, and diseases (34). In human patients, it is related to tiredness, abnormalities in gait, balance (35), and large bone fracture (36, 37) as well as with cognitive impairments (38, 39). Pathological processes caused by acute hyponatremia are well-known, as well as neurological consequences such as brain edema and hyponatremic encephalopathy (40, 41). However, the mechanisms of chronic hyponatremia acting on cognition are poorly understood, although human patients with chronic low sodium levels produced worse scores in cognitive tests (35). Some studies indicate that both chronic and acute hyponatremia in elderly human patients correlate with cognitive decline (35, 41). In veterinary patients, hyponatremia is usually associated with renal disorders, dehydration, vomiting, and diarrhea, and has great impact on the general condition of dogs and cats (42). Thus, in our study we show for the first time a possible correlation between decreased sodium level in blood serum with cognitive impairment in dogs, which may also lead to hypochloremia.

For human neurodegenerative diseases, several biomarkers have been identified in both blood and CSF (43). Although none of them has been used routinely in clinic diagnostics, they can help to predict particular diseases as well as monitor therapeutic efficacy. Similarly, in veterinary medicine, plasma Aβ42 has been considered as a biomarker potentially linked to CCDS in dogs (14).

Based on these studies, we decided to analyze three potential blood biomarkers for dog neurodegeneration, namely, NFL, TAU protein, and Aβ-42 peptide. NFL and TAU proteins are mainly expressed in neurons, while Aβ-42 is formed by amyloid precursor protein (APP). NFL is considered to be a marker for neuronal injury, particularly in myelinated axons of subcortical regions (44) occurring in almost all neurodegenerative diseases (45). In AD, TAU protein undergoes pathological changes and is accumulated in neurofibrillary tangles, while Aβ-42 peptide accumulates in amyloid plaques, so both are the main hallmarks of AD. In this study, we demonstrate for the first time that NFL was increased in senior dogs. We also found that YC presented low levels of NFL, which were increased more than 7-fold in NA dogs. Furthermore, in MiCI dogs, the NFL levels showed 3-fold increase compared to normal-aging controls.

These findings are similar to those in humans, where levels of NFL increase during normal aging and are significantly elevated by neurodegenerative processes in Alzheimer's, Huntington's, or Parkinson's disease (46). Interestingly, levels of NFL in dog serum (NA vs. MiCI; 30.2 vs. 103.3 pg/ml) were very similar to those in human patients with small subcortical infarcts (SI) and were significantly increased compared to healthy controls (HC) (SI vs. HC; 73.45 vs. 34.59 pg/ml, *p* < 0.0001, Simoa) (47). On the other hand, levels of TAU protein and Aβ-42 peptide in serum could not serve as biomarkers for either neurodegeneration or monitoring of dog aging, because their levels hardly changed between all tested groups. This observation correlates with knowledge about levels of TAU and Aβ-42 peptide in human plasma in neurodegeneration, where increases in concentration are only marginal (TAU protein) or are not changed at all (Aβ-42 peptide). Because there are still contradictions in the data as to whether these blood biomarkers can predict cognitive impairment, more studies need to be performed (48).

Dogs suffering from CCDS present several macroscopic and microscopic neuropathological features in the brain, which are similar to those in humans with AD (3, 24, 49–51). One of the similarities is the presence of amyloid plaques (identified with 4G8 antibody), which are mainly found in diffuse and dense forms in CCDS dogs (24, 49, 52). These observations were also confirmed in our study. Furthermore, the presence of amyloid deposits is age dependent, and the process starts at the age of 8 years and develops with age. However, density of plaques varies from case to case (53). In addition, some studies declare direct correlation between the extent of β-amyloid deposits and decline in some cognitive functions (54–57). These data may be at least partially correlated with our findings.

Another important player in the neurodegeneration process is neuroinflammation and microglia behavior (58). Interestingly, our study revealed an activated microglia phenotype with amoeboid body and dystrophic processes, in some cases with spheroidal and bulbous swellings, which is not typically seen in normal aging or young individuals. These changes in dog microglia are very similar to the microglia in humans suffering from neurodegenerative diseases, which is in agreement with our previous study (24). On the other hand, although TAU pathology was found in brains in different mammalian species such as monkeys, ruminants (goat, sheep, reindeers, bison), rodents (59), and also in cats (60), it is quite rare to find it in the aged and CCDS dog population (24). In our study, we did not observe any individuals positive for TAU pathology, and these results are in agreement with our previous work, where we observed TAU pathology only in 1 case out of 28 examined dogs with higher CADES scores (24). Taken together and based on our results from IHC analysis, CCDS dogs partly recapitulate the aging process and early stages of AD pathology. Furthermore, our data are in agreement with other studies suggesting that the blood serum concentration of the NFL might be considered as a biomarker for screening of neurodegenerative disorders also in veterinary medicine.

## Conclusions

Our combination of CADES questionnaires, peripheral biomarkers (ALT, AST, sodium, and chloride levels), and the central biomarker for neurodegeneration (NFL) in canine blood serum seems to be a suitable diagnostic approach for identification of senior dogs with initial CCDS. Furthermore, senior dogs may be considered as a good translation model for studying the aging process and early AD pathology but cannot be fully considered as a model for studying the whole complex of human AD stages.

## Data Availability Statement

The raw data supporting the conclusions of this article will be made available by the authors, without undue reservation.

## Ethics Statement

The animal study was reviewed and approved by the Scientific and Clinical Review Board of UVMPH, University of Veterinary Medicine and Pharmacy in Kosice, Slovakia. The study was carried in accordance with the European Communities Council Directive (2010/63/EU) regarding the use of animals in research and Slovak Law for Animal Protection 377/2012 and 436/2012. Written informed consent was obtained from the owners for the participation of their animals in this study, which was provided by best practice veterinary care.

## Author Contributions

DC and JF: conceptualization. ZV, JF, JH, TS, FH, MM, NZ, LH, and NH: methodology. ZV, JF, JH, TS, FH, MM, and NH: software. DC, ZV, JF, JH, TS, FH, MM, LH, and NZ: writing—original draft preparation. DC, ZV, JF, JH, and TS: writing—review and editing and funding acquisition. All authors have reviewed, read, and approved the manuscript.

## Conflict of Interest

The authors declare that the research was conducted in the absence of any commercial or financial relationships that could be construed as a potential conflict of interest.

## References

[1] Szabó D Gee NR Miklósi Á Natural or pathologic? Discrepancies in the study of behavioral and cognitive signs in aging family dogs. J Vet Behav. (2016) 11:86–98. 10.1016/j.jveb.2015.08.003

[2] Mazzatenta A Carluccio A Robbe D Giulio CD Cellerino A. The companion dog as a unique translational model for aging. Semin Cell Dev Biol. (2017) 70:141–53. 10.1016/j.semcdb.2017.08.024 28803893

[3] Head E. A canine model of human aging and Alzheimer's disease. Biochim Biophys Acta. (2013) 1832:1384–9. 10.1016/j.bbadis.2013.03.016 23528711 PMC3937962

[4] Ruehl WW Bruyette DS dePaoli A Cotman CW Head E Milgram NW Chapter 22 Canine cognitive dysfunction as a model for human age-related cognitive decline, dementia and Alzheimer's disease: clinical presentation, cognitive testing, pathology and response to 1-deprenyl therapy. In: Yu PM, Tipton KF, Boulton AA, editors. Brain Research. Amsterdam; New York, NY: Elsevier (2011). p. 217–25. 10.1016/S0079-6123(08)61218-2 8584657

[5] Neilson JC Hart BL Cliff KD Ruehl WW. Prevalence of behavioral changes associated with age-related cognitive impairment in dogs. J Am Vet Med Assoc. (2001) 218:1787–91. 10.2460/javma.2001.218.1787 11394831

[6] Azkona G García-Belenguer S Chacón G Rosado B León M Palacio J. Prevalence and risk factors of behavioural changes associated with age-related cognitive impairment in geriatric dogs. J Small Anim Pract. (2009) 50:87–91. 10.1111/j.1748-5827.2008.00718.x 19200264

[7] Rosado B González-Martínez Á Pesini P García-Belenguer S Palacio J Villegas A Effect of age and severity of cognitive dysfunction on spontaneous activity in pet dogs – Part 2: social responsiveness. Vet J. (2012) 194:196–201. 10.1016/j.tvjl.2012.03.023 22578689

[8] Madari A Farbakova J Katina S Smolek T Novak P Weissova T Assessment of severity and progression of canine cognitive dysfunction syndrome using the CAnine DEmentia Scale (CADES). Appl Anim Behav Sci. (2015) 171:138–45. 10.1016/j.applanim.2015.08.034

[9] Osella MC Re G Odore R Girardi C Badino P Barbero R Canine cognitive dysfunction syndrome: prevalence, clinical signs and treatment with a neuroprotective nutraceutical. Appl Anim Behav Sci. (2007) 105:297–310. 10.1016/j.applanim.2006.11.007

[10] Landsberg GM Nichol J Araujo JA. Cognitive dysfunction syndrome: a disease of canine and feline brain aging. Vet Clin. (2012) 42:749–68. 10.1016/j.cvsm.2012.04.003 22720812

[11] Fast R Schütt T Toft N Møller A Berendt M. An observational study with long-term follow-up of canine cognitive dysfunction: clinical characteristics, survival, risk factors. J Vet Intern Med. (2013) 27:822–9. 10.1111/jvim.12109 23701137

[12] Pugliese M Carrasco JL Andrade C Mas E Mascort J Mahy N Severe cognitive impairment correlates with higher cerebrospinal fluid levels of lactate and pyruvate in a canine model of senile dementia. Prog Neuro Psychopharmacol Biol Psychiatry. (2005) 29:603–10. 10.1016/j.pnpbp.2005.01.017 15866364

[13] González-Martínez Á Rosado B Pesini P Suárez M.-L. Santamarina G. Plasma β-amyloid peptides in canine aging and cognitive dysfunction as a model of Alzheimer's disease. Exp Gerontol. (2011) 46:590–6. 10.1016/j.exger.2011.02.013 21377518

[14] Schütt T Toft N Berendt M A comparison of 2 screening questionnaires for clinical assessment of canine cognitive dysfunction. J Vet Behav. (2015) 10:452–8. 10.1016/j.jveb.2015.07.036

[15] Borghys H van Broeck B Dhuyvetter D Jacobs T de Waepenaert K Erkens T. Young to middle-aged dogs with high amyloid-β levels in cerebrospinal fluid are impaired on learning in standard cognition tests. J Alzheimers Dis. (2017) 56:763–74. 10.3233/JAD-160434 28035921 PMC5271428

[16] O'Bryant SE Mielke MM Rissman RA Lista S Vanderstichele H Zetterberg H. Blood-based biomarkers in Alzheimer disease: current state of the science and a novel collaborative paradigm for advancing from discovery to clinic. Alzheimer's Dement. (2017) 13:45–58. 10.1016/j.jalz.2016.09.014 27870940 PMC5218961

[17] Preische O Schultz SA Apel A Kuhle J Kaeser SA Barro C. Serum neurofilament dynamics predicts neurodegeneration and clinical progression in presymptomatic Alzheimer's disease. Nat Med. (2019) 25:277–83. 10.1038/s41591-018-0304-3 30664784 PMC6367005

[18] Tapp PD Siwak CT Gao FQ Chiou J.-Y. Black SE. Frontal lobe volume, function, and β-amyloid pathology in a canine model of aging. J Neurosci. (2004) 24:8205–13. 10.1523/JNEUROSCI.1339-04.2004 15385603 PMC6729694

[19] Siwak-Tapp CT Head E Muggenburg BA Milgram NW Cotman CW. Region specific neuron loss in the aged canine hippocampus is reduced by enrichment. Neurobiol Aging. (2008) 29:39–50. 10.1016/j.neurobiolaging.2006.09.018 17092609 PMC2198929

[20] Preston JE. Ageing choroid plexus-cerebrospinal fluid system. Microsc Res Tech. (2001) 52:31–7. 10.1002/1097-0029(20010101)52:1<31::AID-JEMT5>3.0.CO;2-T 11135446

[21] Vite CH Head E. Aging in the canine and feline brain. Vet Clin North Am. (2014) 44:1113–29. 10.1016/j.cvsm.2014.07.008 25441628 PMC4254595

[22] Schmidt F Boltze J Jager C Hofmann S Willems N Seeger J. Detection and quantification of A-Amyloid, Pyroglutamyl AA, and Tau in aged canines. J. Neuropathol Exp Neurol. (2015) 74:12. 10.1097/NEN.0000000000000230 26247394

[23] Rusbridge C Salguero FJ David MA Faller KME Bras JT Guerreiro RJ. An aged canid with behavioral deficits exhibits blood and cerebrospinal fluid amyloid beta oligomers. Front Aging Neurosci. (2018) 10:7. 10.3389/fnagi.2018.00007 29441010 PMC5797595

[24] Smolek T Madari A Farbakova J Kandrac O Jadhav S Cente M. Tau hyperphosphorylation in synaptosomes and neuroinflammation are associated with canine cognitive impairment: synaptic tau in canine dementia. J Comp Neurol. (2016) 524:874–95. 10.1002/cne.23877 26239295

[25] Johnstone EM Chaney MO Norris FH Pascual R Little SP. Conservation of the sequence of the Alzheimer's disease amyloid peptide in dog, polar bear and five other mammals by cross-species polymerase chain reaction analysis. Brain Res Mol Brain Res. (1991) 10:299–305. 10.1016/0169-328X(91)90088-F 1656157

[26] Alafuzoff I Thal DR Arzberger T Bogdanovic N Al-Sarraj S Bodi I. Assessment of β-amyloid deposits in human brain: a study of the BrainNet Europe consortium. Acta Neuropathol. (2009) 117:309–20. 10.1007/s00401-009-0485-4 19184666 PMC2910889

[27] Sachdev PS Blacker D Blazer DG Ganguli M Jeste DV Paulsen JS. Classifying neurocognitive disorders: the DSM-5 approach. Nat. Rev. Neurol. (2014) 10:634–42. 10.1038/nrneurol.2014.181 25266297

[28] Ozawa M Chambers JK Uchida K Nakayama H. The relation between canine cognitive dysfunction and age-related brain lesions. J Vet Med Sci. (2016) 78:997–1006. 10.1292/jvms.15-0624 26922972 PMC4937160

[29] Takeuchi T Harada E. Age-related changes in sleep-wake rhythm in dog. Behav. Brain Res. (2002) 136:193–9. 10.1016/S0166-4328(02)00123-7 12385805

[30] Sattar N Scherbakova O Ford I O'Reilly DSJ Stanley A Forrest E. Elevated alanine aminotransferase predicts new-onset type 2 diabetes independently of classical risk factors, metabolic syndrome, and C-reactive protein in the west of Scotland coronary prevention study. Diabetes. (2004) 53:2855–60. 10.2337/diabetes.53.11.2855 15504965

[31] Goessling W Massaro JM Vasan RS D'Agostino RB Ellison RC Fox CS. Aminotransferase levels and 20-year risk of metabolic syndrome, diabetes, cardiovascular disease. Gastroenterology. (2008) 135:1935–44.e1. 10.1053/j.gastro.2008.09.018 19010326 PMC3039001

[32] Nho K Kueider-Paisley A Ahmad S MahmoudianDehkordi S Arnold M Risacher SL. Association of altered liver enzymes with alzheimer disease diagnosis, cognition, neuroimaging measures, and cerebrospinal fluid biomarkers. JAMA Netw Open. (2019) 2:e197978. 10.1001/jamanetworkopen.2019.7978 31365104 PMC6669786

[33] Estrada LD Ahumada P Cabrera D Arab JP. Liver dysfunction as a novel player in alzheimer's progression: looking outside the brain. Front Aging Neurosci. (2019) 11:174. 10.3389/fnagi.2019.00174 31379558 PMC6650779

[34] Filippatos TD Makri A Elisaf MS Liamis G. Hyponatremia in the elderly: challenges and solutions. CIA Volume. (2017) 12:1957–65. 10.2147/CIA.S138535 29180859 PMC5694198

[35] Renneboog B Musch W Vandemergel X Manto MU Decaux G. Mild chronic hyponatremia is associated with falls, unsteadiness, attention deficits. Am J Med. (2006) 119:71.e1–8. 10.1016/j.amjmed.2005.09.026 16431193

[36] Gankam Kengne F Andres C Sattar L Melot C Decaux G. Mild hyponatremia and risk of fracture in the ambulatory elderly. QJM. (2008) 101:583–88. 10.1093/qjmed/hcn061 18477645

[37] Sandhu HS Gilles E DeVita MV Panagopoulos G Michelis MF. Hyponatremia associated with large-bone fracture in elderly patients. Int Urol Nephrol. (2009) 41:733–7. 10.1007/s11255-009-9585-2 19472069

[38] Gunathilake R Oldmeadow C McEvoy M Kelly B Inder K Schofield P. Mild hyponatremia is associated with impaired cognition and falls in community-dwelling older persons. J Am Geriatr Soc. (2013) 61:1838–9. 10.1111/jgs.12468 24117308

[39] Fujisawa H Sugimura Y Takagi H Mizoguchi H Takeuchi H Izumida H. Chronic hyponatremia causes neurologic and psychologic impairments. JASN. (2016) 27:766–80. 10.1681/ASN.2014121196 26376860 PMC4769197

[40] Ayus JC Arieff AI. Pathogenesis and prevention of hyponatremic encephalopathy. Endocrinol Metab Clin North Am. (1993) 22:425–46. 10.1016/S0889-8529(18)30175-0 8325296

[41] Soiza RL Cumming K Clarke JM Wood KM Myint PK. Hyponatremia: special considerations in older patients. J Clin Med. (2014) 3:944–58. 10.3390/jcm3030944 26237487 PMC4449639

[42] Ueda Y Hopper K Epstein SE. Incidence, severity and prognosis associated with hyponatremia in dogs and cats. J Vet Intern Med. (2015) 29:801–7. 10.1111/jvim.12581 25996661 PMC4895431

[43] McKhann GM Knopman DS Chertkow H Hyman BT Jack CR Kawas CH The diagnosis of dementia due to Alzheimer's disease: recommendations from the National institute on aging-Alzheimer's association workgroups on diagnostic guidelines for Alzheimer's disease. Alzheimers Dement. (2011) 7:263–9. 10.1016/j.jalz.2011.03.005 21514250 PMC3312024

[44] Zetterberg H Smith DH Blennow K. Biomarkers of mild traumatic brain injury in cerebrospinal fluid and blood. Nat. Rev. Neurol. (2013) 9:201–10. 10.1038/nrneurol.2013 23399646 PMC4513656

[45] Lewczuk P Ermann N Andreasson U Schultheis C Podhorna J Spitzer P. Plasma neurofilament light as a potential biomarker of neurodegeneration in Alzheimer's disease. Alzheimers Res Ther. (2018) 10:71. 10.1186/s13195-018-0404-9 30055655 PMC6064615

[46] Byrne LM Rodrigues FB Blennow K Durr A Leavitt BR Roos RAC. Neurofilament light protein in blood as a potential biomarker of neurodegeneration in Huntington's disease: a retrospective cohort analysis. Lancet Neurol. (2017) 16:601–9. 10.1016/S1474-4422(17)30124-2 28601473 PMC5507767

[47] Gattringer T Pinter D Enzinger C Seifert-Held T Kneihsl M Fandler S Serum neurofilament light is sensitive to active cerebral small vessel disease. Neurology. (2017) 89:2108–14. 10.1212/WNL.0000000000004645 29046363 PMC5711505

[48] Chen T-B Lee Y-J Lin S-Y Chen J-P Hu C-J Wang P-N. Plasma Aβ42 and total tau predict cognitive decline in amnestic mild cognitive impairment. Sci Rep. (2019) 9:13984. 10.1038/s41598-019-50315-9 31562355 PMC6764975

[49] Cummings BJ Su JH Cotman CW White R Russell MJ. Beta-amyloid accumulation in aged canine brain: a model of early plaque formation in Alzheimer's disease. Neurobiol Aging. (1993) 14:547–60. 10.1016/0197-4580(93)90038-D 8295657

[50] Kimotsuki T Yasuda M Tamahara S Tomihari M Matsuki N Ono K. Age-associated changes of flash visual evoked potentials in dogs. J Vet Med Sci. (2006) 68:79–82. 10.1292/jvms.68.79 16462123

[51] Pugliese M Geloso MC Carrasco JL Mascort J Michetti F Mahy N. Canine cognitive deficit correlates with diffuse plaque maturation and S100β (–) astrocytosis but not with insulin cerebrospinal fluid level. Acta Neuropathol. (2006) 111:519–28. 10.1007/s00401-006-0052-1 16718348

[52] Insua D Suárez M-L Santamarina G Sarasa M Pesini P. Dogs with canine counterpart of Alzheimer's disease lose noradrenergic neurons. Neurobiol Aging. (2010) 31:625–35. 10.1016/j.neurobiolaging.2008.05.014 18573571

[53] Czasch S Paul S Baumgärtner W. A comparison of immunohistochemical and silver staining methods for the detection of diffuse plaques in the aged canine brain. Neurobiol Aging. (2006) 27:293–305. 10.1016/j.neurobiolaging.2005.02.017 16002188

[54] Cummings BJ Head E Ruehl W Milgram NW Cotman CW. The canine as an animal model of human aging and dementia. Neurobiol Aging. (1996) 17:259–68. 10.1016/0197-4580(95)02060-8 8744407

[55] Colle M-A Hauw J-J Crespeau F Uchihara T Akiyama H Checler F Pageat P. Vascular and parenchymal Aβ deposition in the aging dog: correlation with behavior. Neurobiol Aging. (2000) 21:695–704. 10.1016/S0197-4580(00)00113-5 11016539

[56] Pugliese M Mascort J Mahy N Ferrer I. Diffuse beta-amyloid plaques and hyperphosphorylated tau are unrelated processes in aged dogs with behavioral deficits. Acta Neuropathol. (2006) 112:175–83. 10.1007/s00401-006-0087-3 16775693

[57] Rofina JE van Ederen AM Toussaint MJM Secrève M van der Spek A van der Meer I. Cognitive disturbances in old dogs suffering from the canine counterpart of Alzheimer's disease. Brain Res. (2006) 1069:216–26. 10.1016/j.brainres.2005.11.021 16423332

[58] Rofina J van Andel I van Ederen AM Papaioannou N Yamaguchi H Gruys E. Canine counterpart of senile dementia of the Alzheimer type: amyloid plaques near capillaries but lack of spatial relationship with activated microglia and macrophages. Amyloid. (2003) 10:86–96. 10.3109/13506120309041730 12964416

[59] Härtig W Klein C Brauer K Schüppel KF Arendt T Brückner G. Abnormally phosphorylated protein tau in the cortex of aged individuals of various mammalian orders. Acta Neuropathol. (2000) 100:305–12. 10.1007/s004010000183 10965801

[60] Fiock KL Smith JD Crary JF Hefti MM. β-amyloid and tau pathology in the aging feline brain. J Comp Neurol. (2020) 528:108–13. 10.1002/cne.24741 31273784 PMC6842105

