# Erratum: Novel Diagnostic Tools for Identifying Cognitive Impairment in Dogs: Behavior, Biomarkers, and Pathology

**DOI:** 10.3389/fvets.2021.658344

**Published:** 2021-02-11

**Authors:** Frontiers Production Office

**Affiliations:** Frontiers Media SA, Lausanne, Switzerland

**Keywords:** canine cognitive dysfunction, neurodegeneration, CADES, questionnaire biomarkers, TAU, Aβ42, NFL

Due to a production error, there was a mistake in Figure 6 as published. Two extra rows were mistakenly included. The corrected Figure 6 appears below.

**Figure 6:**
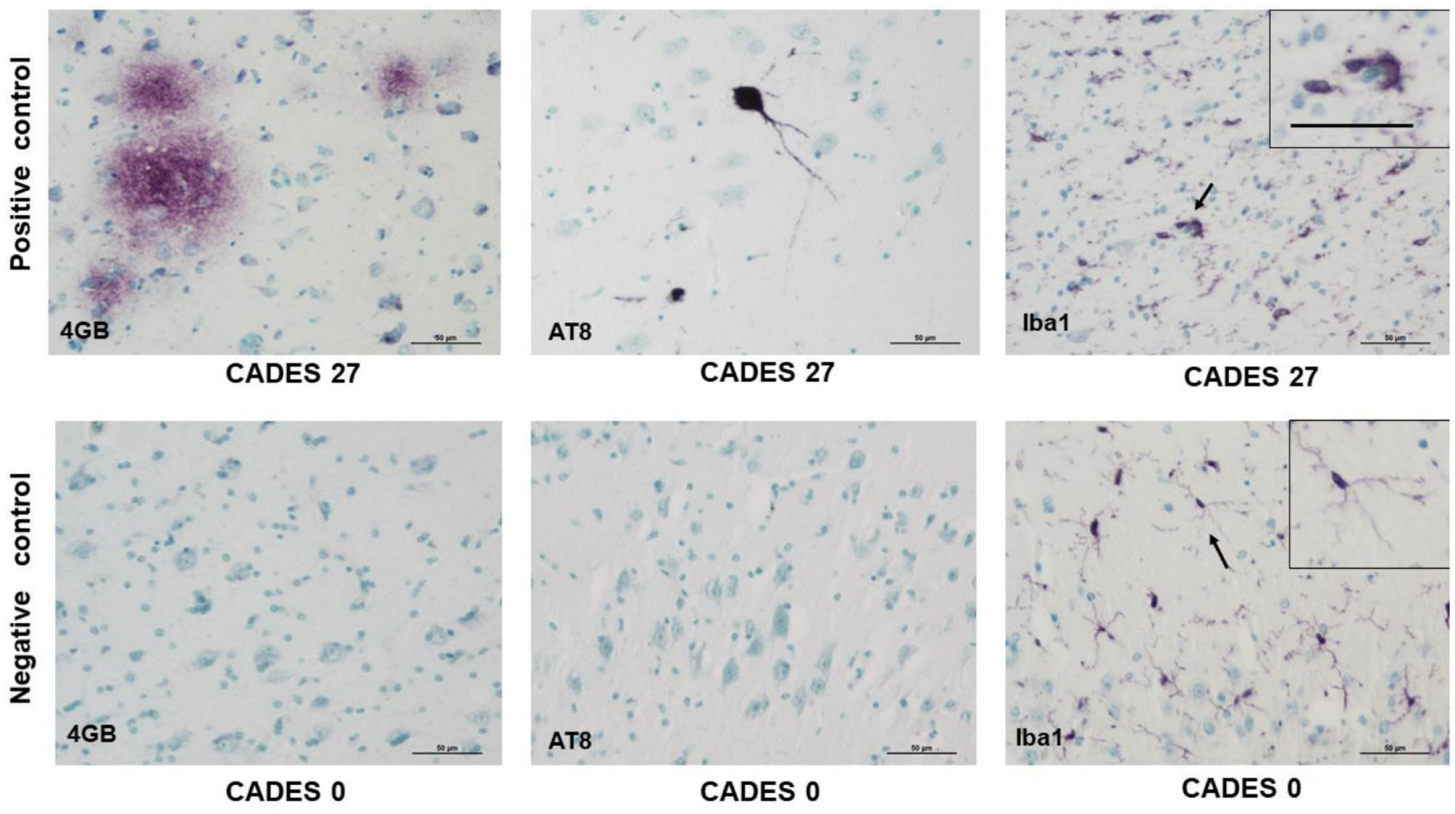
Immunohistochemistry of cortex in positive control/dog with cognitive declaim (CADES 27) and negative control/dog with normal cognition (CADES 0) showed differences in all three antibodies. While in positive control, we could identify amyloid deposits (4GB), occasional TAU (AT8) pathology, and activated microglia (Iba1), with amoeboid morphology (indicated by arrow), cortex of negative control was free of amyloid and TAU. Resting microglia with fine, ramified processes was detected (indicated by arrow). Note, inserts in positive/negative control with higher magnification of microglia morphology. Scale bars: 50 μm.

Additionally, due to a production error, the funding number for the funder APVV, was erroneously omitted. The missing number is “APVV-19-0193 (DC)”.

The publisher apologizes for these mistakes. The original article has been updated.

