# Supplementary material for: Novel Diagnostic Tools for Identifying Cognitive Impairment in Dogs: Behavior, Biomarkers, and Pathology
Source: Front Vet Sci. 2021 Jan 15;7:551895. doi: 10.3389/fvets.2020.551895 (PMC7843503; doi:10.3389/fvets.2020.551895)
Supplement: Supplementary file 1 [file Table_1.DOCX]

| **DOMAINS/ITEMS** | **FREQUENCY** |
| --- | --- |
|  |  |
| **A. SPATIAL ORIENTATION** |  |
| **1**, disorientation in a familiar environment (inside/outside) |  |
| **2**, recognition familiar people and animals inside or outside the house/apartment |  |
| **3**, abnormally respond to familiar object (a chair, a wastebasket) |  |
| **4**, aimlessly wandering (motorically restless during day) |  |
| **5**, reduced ability to do previously learned tasks |  |
|  |  |
| **SCORE(0-25)** |  |
|  |  |
| **B. SOCIAL INTERACTION** |  |
| **6**, changes in interaction a man/dog, dog/other dog (playing, petting, welcoming) |  |
| **7**, changes in individual behaviour of dog (exploration behaviour, play, performance) |  |
| **8**, response to commands and ability to learn new tasks |  |
| **9**, irritable |  |
| **10**, expression of aggression |  |
|  |  |
| **SCORE (0-25)** |  |
|  |  |
| **C. SLEEP-WAKE CYCLES** |  |
| **11**, abnormally responds in night (wandering, vocalization, motirically restless) |  |
| **12,** switch over from insomnia to hypersomnia |  |
|  |  |
| **SCOREx2 (0-20)** |  |
|  |  |
| **D. HOUSE SOILING** |  |
| **13**, eliminate at home at random locations |  |
| **14**, eliminate in its kennel or sleeping area |  |
| **15**, changes in signalisation for elimination activity |  |
| **16**, eliminate indoors after a recent walk outside |  |
| **17**, eliminate at uncommon locations (grass, concentrate) |  |
| **SCORE(0-25)** |  |
|  |  |
| **TOTAL SCORE (A+B+C+D) (0-95)** |  |
| **CLINICAL STAGES:**  **Normal ageing (Score 0 – 7), Mild cognitive impairment (8 – 23), Moderate cognitive impairment (24 – 44), Cognitive dysfunction (45 – 95)** |  |
|  |  |
|  |  |
| **Name of dog: Age: Sex: Breed:** |  |
| **Housing (outside/inside): Food (pellets/mix):** |  |
| **Castration:** |  |

**Canine Dementia Scale (CADES)**

Frequency:

0 – abnormal behaviour of the dog was never observed

2 – abnormal behaviour of the dog was detected at least once in the last 6 months

3 – abnormal behaviour of the dog appeared at least once per month

4 – abnormal behaviour of the dog was seen 2-4 times per month

5 – abnormal behaviour of the dog was observed several times a week
